# Effective comparative analysis of protein-protein interaction networks by measuring the steady-state network flow using a Markov model

**DOI:** 10.1186/s12859-016-1215-2

**Published:** 2016-10-06

**Authors:** Hyundoo Jeong, Xiaoning Qian, Byung-Jun Yoon

**Affiliations:** Department of Electrical and Computer Engineering, Texas A&M University, College Station, USA

## Abstract

**Background:**

Comparative analysis of protein-protein interaction (PPI) networks provides an effective means of detecting conserved functional network modules across different species. Such modules typically consist of orthologous proteins with conserved interactions, which can be exploited to computationally predict the modules through network comparison.

**Results:**

In this work, we propose a novel probabilistic framework for comparing PPI networks and effectively predicting the correspondence between proteins, represented as network nodes, that belong to conserved functional modules across the given PPI networks. The basic idea is to estimate the steady-state network flow between nodes that belong to different PPI networks based on a Markov random walk model. The random walker is designed to make random moves to adjacent nodes within a PPI network as well as cross-network moves between potential orthologous nodes with high sequence similarity. Based on this Markov random walk model, we estimate the steady-state network flow – or the long-term relative frequency of the transitions that the random walker makes – between nodes in different PPI networks, which can be used as a probabilistic score measuring their potential correspondence. Subsequently, the estimated scores can be used for detecting orthologous proteins in conserved functional modules through network alignment.

**Conclusions:**

Through evaluations based on multiple real PPI networks, we demonstrate that the proposed scheme leads to improved alignment results that are biologically more meaningful at reduced computational cost, outperforming the current state-of-the-art algorithms. The source code and datasets can be downloaded from http://www.ece.tamu.edu/~bjyoon/CUFID.

## Background

Complex biological mechanisms such as signaling pathways and metabolic processes are governed and coordinated by numerous protein-protein interactions (PPIs). In addition to gene expression profiles, PPIs provide invaluable information that can be exploited to predict novel functional modules that perform critical biological functions. Thanks to recent advances in high-throughput protein interaction measurement techniques, PPI networks for different species have been archived in public databases, where the coverage and quality of these networks continue to improve over time. To translate these protein interaction data into useful biological knowledge – for example, that of the functional organization of cells and the detailed mechanisms of various cellular functions – we need effective means for analyzing the available PPI networks to accurately annotate the protein functions and to identify modules of proteins that may be potentially involved in crucial biological processes conserved across species.

Although one can study functions of proteins in PPI networks through biological experiments, it takes a large amount of valuable resources including labor, experimental cost, and time. As we have increasing evidence that various functional modules are conserved across different species, in which orthologous proteins and their interactions are preserved, comparative network analysis based on computational approaches would be one reasonable alternative that can save the cost and time of expensive biological experiments [1, 2]. Through comparative network analysis techniques such as network querying and network alignment [3], we can identify conserved functional modules as well as functionally similar proteins. By identifying corresponding protein nodes across networks, functional annotations of known proteins in well-studied species could be transferred to matching proteins in the PPI networks of less-studied species, which provides an efficient way of predicting potential functions of unknown proteins.

**Fig. 1:**
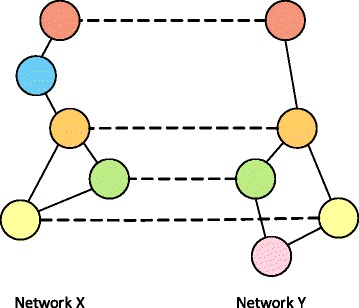
Illustration of a pairwise network alignment. The *blue colored* node in network X is deleted in network Y, and the *pink colored* node in network Y is inserted. Note that an inserted node in one network can be viewed as a deleted node in the other network

To obtain biologically meaningful PPI network alignment results, we should take both the molecular-level similarity between proteins as well as the similarity of their interaction patterns into account. The pairwise molecular-level similarity can be measured by comparing the sequence (or structure) of the proteins. As shown in [1, 2], interactions between orthologous proteins are often well-preserved in functional modules that are commonly found in multiple species. As a result, it would be desirable to consider the topological similarity between PPI networks, which arises from such conserved PPIs, for accruately comparing and aligning networks. Hence, an essential first step to construct a reliable network alignment is to accurately estimate the universal similarity measure that reflects the *node correspondence* across networks by integrating the two types of similarities: pairwise node similarity and topological similarity. However, several factors make the estimation of the node correspondence practically difficult. First, when comparing PPI networks of different species, not all protein nodes are present in all PPI networks, hence the networks are bound to have a large number of inserted/deleted nodes (see Fig. 1). Second, the interaction patterns may significantly vary in different PPI networks, where orthologous proteins in different species may interact with considerably different sets of proteins in the respective networks. As a result, the PPI networks may have a large number of inserted/deleted edges. Third, most nodes may have numerous potential matching nodes in other networks. All these factors make accurate prediction of node correspondence quite challenging.

Several network alignment algorithms have been proposed to identify and predict orthologous protein pairs and conserved functional modules in different networks. The pioneering network alignment algorithms, PathBLAST [4] and NetworkBLAST [1, 5], focus on identifying highly conserved local complexes. However, PathBLAST can only search for linear paths, and NetworkBLAST constructs local alignments where one protein can have multiple matching partners, which may yield ambiguous alignments. IsoRank [6] estimates the node correspondence using a modification of the widely-known PageRank algorithm [7], where the basic idea is that two proteins have a high probability to be aligned if their neighboring proteins are also matched well. IsoRankN [8] extends IsoRank to align multiple PPI networks by adopting PageRank-Nibble [9], a spectral clustering method. IsoRank and IsoRankN are relatively time consuming and require a huge amount of memory as the size of the network increases. SMETANA [10] adopts a semi-Markov random walk (SMRW) model to estimate the node correspondence scores. These scores are updated through the intra-network and cross-network probabilistic consistency transformations, which are subsequently used to greedily build the network alignment. SMETANA-CSRW [11] estimates the node correspondence scores using a context-sensitive random walk (CSRW) model [12], which integrates the node similarity and the topological similarity between networks. Then, it constructs the final alignment based on a greedy approach. Although SMETANA-CSRW has slightly higher computational complexity as the network size increases, the utilization of the CSRW model has been shown to improve the accuracy of the alignment results. PINALOG [13] detects dense subnetworks as communities. Then, it constructs the initial community mapping and extends the alignment by mapping the neighboring nodes of the core proteins. HubAlign [14] first assigns weights to the nodes and edges in the PPI networks based on their topological importance (i.e., likelihood to be a hub), and then calculates the alignment score for every pair of proteins based on the global topological property and sequence information. Then, the algorithm constructs a global network alignment using a greedy seed-and-extension approach. Both PINALOG and HubAlign are more dependent on the topological similarity between networks than node similarity for obtaining the network alignment results, which may degrade the alignment accuracy when handling incomplete PPI networks or networks that may contain a relatively large number of false positive interactions.

In this paper, we propose a novel network alignment algorithm, called **CUFID-align** (**C**omparative network analysis **U**sing the steady-state network **F**low to **ID**entify orthologous proteins). The algorithm estimates the node correspondence by measuring the steady-state network flow of a random walk model over an *integrated network* of the given PPI networks. To accurately estimate the node correspondence based on the steady-state network flow, in a way that effectively captures the biological significance, we design the Markov random walk model such that the relative frequency that the random walker makes transitions between a pair of nodes in different PPI networks is proportional to the pairwise node similarity and the topological similarity between the surrounding network regions. The proposed scheme effectively captures the functional correspondence between nodes across different networks and the estimated node correspondence scores can lead to accurate network alignment results, as will be demonstrated through performance assessment based on real PPI networks.

## Methods

### Problem formulation

Suppose that we have a pair of PPI networks with the graph representations ${\mathcal {G}}_{X} = \left ({{\mathcal {U}},{\mathcal {D}}}\right)$ and ${\mathcal {G}}_{Y} = \left ({{\mathcal {V}},{\mathcal {E}}} \right)$, in which nodes represent proteins in each PPI network (i.e., $u_{i} \in \mathcal {U}$ or $v_{j} \in \mathcal {V}$), and edges ($d_{ij} \in \mathcal {D}$ or $e_{ij} \in \mathcal {E}$) indicate that the corresponding protein *u*_*i*_ (or *v*_*i*_) binds with the protein *u*_*j*_ (or *v*_*j*_). The edge weights in the PPI networks can indicate the strength or confidence of the interactions between the proteins. Given a pair of nodes across the PPI networks, we assume that the pairwise node similarity score *s*(*u*_*i*_,*v*_*j*_), $u_{i} \in {\mathcal {U}}$ and $v_{j} \in {\mathcal {V}}$ can be computed, for example, based on the sequence similarity between the proteins. In this study, we utilized BLAST bit scores between proteins as the pairwise node similarity scores. However, other types of similarity measurements (or their combinations) could be also used as the pairwise node similarity score in case such measurements can be easily obtained.

Given a pair of PPI networks ${\mathcal {G}}_{X}$ and ${\mathcal {G}}_{Y}$, our objective is to derive the optimal one-to-one mapping *A* ^∗^ between nodes in different PPI networks. One possible criterion that could be used to find such a mapping is the maximum expected accuracy (MEA) criterion, which aims to maximize the expected number of correctly mapped nodes. Provided that we can derive a pairwise node alignment probability $ \Pr \left [{u_{i}\sim v_{j}|{\mathcal {G}}_{X},{\mathcal {G}}_{Y}}\right ]$, $u_{i}\in {\mathcal {U}}$ and $v_{j} \in {\mathcal {V}}$, the optimal one-to-one mapping can be found by:

1 $$ A^{*} = \mathop {\arg \max }\limits_{A} \sum\limits_{\forall \left({u_{i} \sim v_{j}}\right) \in A} {\Pr \left[{u_{i} \sim v_{j} |{\mathcal{G}}_{X},{\mathcal{G}}_{Y}}\right]}   $$

according to the MEA criterion. This MEA approach has been widely used by many multiple sequence alignment algorithms [15–19] and it has been shown to be useful for network alignment [10, 11] and network querying [20] as well.

### Motivation and overview of the proposed method

Based on the above problem setting, to construct a confident network alignment, it is crucial to accurately estimate the pairwise node alignment probabilities. To obtain biologically meaningful alignment results, it is necessary that the pairwise node alignment probability is proportional to both the pairwise node similarity (i.e., sequence similarity) and the topological similarity between the subnetwork regions surrounding the nodes in the respective networks. This is based on the observation that orthologous proteins typically have a high level of compositional similarity and often display similar interaction patterns to their neighboring nodes [1, 2]. To accurately estimate the pairwise node alignment probability by effectively integrating these two different types of similarities, we propose to utilize the concept of steady-state network flow (i.e., the amount of ‘water’ that flows through a given channel in the network). Similar concepts have been previously adopted in various engineering applications to find the solutions to similar assignment problems. For example, in digital communication systems, the water-filling algorithm [21] is utilized to compute the optimal allocation of resources. Conceptually, it pours ‘water’ into an OFDM (orthogonal frequency division multiplexing) channel, and the ‘water level’ in the OFDM channel is utilized to find the optimal solution of the transmit power for each subcarrier. In digital image processing, the so-called watershed method [22] is used to find edges or contours of objects in the given image. The watershed method assumes that ‘water’ flows along the image gradient (e.g., intensity differences) and eventually reaches the local minima so that the ‘water level’ in the image provides the solution for the desired image segmentation.

**Fig. 2:**
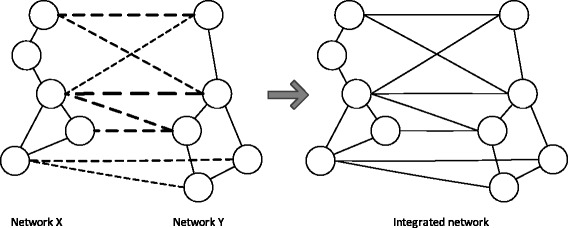
Illustration for constructing the integrated network from a network pair. *Dotted lines* in the left figure indicate the pairwise node similarity, in which the thickness of each line is proportional to the pairwise node similarity. To construct the integrated network, we insert an edge between each pair of nodes across different networks if they have positive pairwise node similarity

**Fig. 3:**
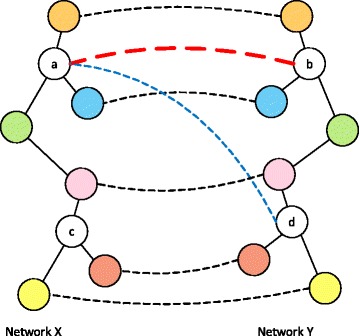
Illustration of how node correspondence is measured based on the steady-state network flow. *Straight lines* represent protein-protein interactions, and *dotted lines* indicate pairwise node similarity. In this example, there is a larger steady-state network flow between the node pair (*a*, *b*) than the node pair (*a*, *d*) because the node pair (*a*, *b*) has higher pairwise node similarity and as the nodes have similar interacting nodes in the respective networks. In contrast, although the node pair (*a*, *d*) has positive pairwise node similarity, the neighboring nodes in the respective networks are not similar, which leads to a smaller steady-state network flow between the nodes (*a*, *d*) compared to the flow between (*a*, *b*)

In the proposed method, we measure the steady-state network flow in an integrated network that is obtained by combining the PPI network pair to be aligned. More specifically, edges are inserted between nodes in different networks that have positive pairwise node similarity, and the pairwise node similarity score is assigned as the edge weight (see Fig. 2). Suppose we pour ‘water’ on the integrated network and that the amount of water flow is proportional to the edge weight. If a given pair of nodes in different PPI networks have higher pairwise node similarity and if their neighboring nodes also have higher pairwise node similarity, there would be a larger water flow between the pair of nodes in the long run. However, if the nodes have a similar topological structure (i.e., in terms of the number of interacting nodes in the respective networks) but if their neighboring nodes are not similar, there will be relatively small water flow between the pair of nodes (see Fig. 3). As a result, the water flow between nodes across different PPI networks provides an intuitive way of measuring the overall similarity of the nodes – or functional correspondence between the proteins. As will be shown later, the resulting node correspondence score obtained based on the concept of water flow in the integrated network can serve as an effective building block for constructing an accurate and biologically meaningful network alignment.

### Estimating the node correspondence through a Markov random walk model

In order to effectively estimate the node correspondence by integrating both the pairwise node similarity and topological similarity using a Markov random walk model, we first construct the integrated network *G*=(*V*,*E*) by combining ${\mathcal {G}}_{X}$ and ${\mathcal {G}}_{Y}$. Nodes of the integrated network *G* are the union of the nodes of ${\mathcal {G}}_{X}$ and ${\mathcal {G}}_{Y}$ (i.e., $V={\left \{ {{\mathcal {U}},{\mathcal {V}}} \right \}}$), and edges are the union of the edges of ${\mathcal {G}}_{X}$, ${\mathcal {G}}_{Y}$, and additional weighted edges ${\mathcal {F}}$, where ${\mathcal {F}} = \left \{ {s\left ({u_{i},v_{j}} \right)|u_{i} \in {\mathcal {U}},v_{j} \in {\mathcal {V}}} \right \}$ (i.e., $E={\left \{ {{\mathcal {D}},{\mathcal {E}},{\mathcal {F}}} \right \}}$). On this integrated network *G*, we allow the random walker to randomly move from the current node to any of its neighboring nodes at each time step. We define two different types of random moves based on their starting and ending points. First, if the random walker moves from a node in ${\mathcal {U}}$ to a node in ${\mathcal {U}}$ (or from a node in ${\mathcal {V}}$ to a node in ${\mathcal {V}}$), we define it as an *intra-network* random move, as the random walk takes place in the same PPI network. Second, if the random walker moves from a node in ${\mathcal {U}}$ to a node in ${\mathcal {V}}$ (or from a node in ${\mathcal {V}}$ to a node in ${\mathcal {U}}$), we refer to this as a *cross-network* random move. The intra-network random move mainly aims to capture the topological similarity between the two PPI networks while the cross-network random move aims to incorporate the pairwise node similarity between nodes that originally belong to different PPI networks.

The transition probabilities of the resulting random walker are determined as follows. Suppose the two networks ${\mathcal {G}}_{X} = \left ({{\mathcal {U}},{\mathcal {D}}}\right)$ and ${\mathcal {G}}_{Y} = \left (\mathcal {V},\mathcal {E}\right)$ have weighted edges, where the respective adjacency matrices are given by:

2a $$\begin{array}{*{20}l} & A_{X} \left[{i,j} \right] = \left\{ \begin{array}{ll} d_{ij}, & \left({u_{i},u_{j}} \right) \in {\mathcal{D}}\\ {0,} & {otherwise} \\ \end{array}, \right. \end{array} $$

2b $$\begin{array}{*{20}l} &A_{Y} \left[i,j \right] = \left\{ \begin{array}{ll} e_{ij}, & \left(v_{i},v_{j}\right) \in {\mathcal{E}}\\ 0, & otherwise \\ \end{array}. \right. \end{array} $$

First of all, to compute the transition probabilities of the intra-network random moves, we transform the edge weighted adjacency matrix into a legitimate stochastic matrix by normalizing each row. That is, the transition probability of the random walker is proportional to the weight of the edge that connects the node at the current position of the random walker and the neighboring node (in the same PPI network) to which it wants to move. The resulting transition probability of any intra-network random move is given by

3 $$ P_{k} \left[i,j \right] = \frac{1}{\sum\limits_{\forall j} A_{k} \left[ {i,j} \right]} \cdot A_{k} \left[ {i,j} \right],k = X,Y.   $$

Eq. (3) can be rewritten in a simple matrix form, which is given by

4 $$ \textbf{P}_{X} = \textbf{D}_{X}^{- 1} \cdot \textbf{A}_{X} \text{ and } \textbf{P}_{Y} = \textbf{D}_{Y}^{- 1} \cdot \textbf{A}_{Y},  $$

where **D**_*X*_ is a $\left | {\mathcal {U}} \right | \times \left | {\mathcal {U}} \right |$ dimensional diagonal matrix such that $D_{X} \left [ {i,i} \right ] = \sum \limits _{\forall j} {A_{X} \left [ {i,j} \right ]} $, and **D**_*Y*_ is a $\left | {\mathcal {V}} \right | \times \left | {\mathcal {V}} \right |$ dimensional diagonal matrix such that $D_{Y} \left [i,i\right ] = \sum \limits _{\forall j} {A_{Y} \left [ {i,j}\right ]}$.

Next, suppose that the transition probability of the cross-network random move between two nodes in different networks is proportional to their pairwise node similarity score. That is, from the current position of the random walker in a given PPI network, the random walker is more likely to move to a node in the other PPI network with higher pairwise node similarity. This will increase the ‘network flow’ between nodes that have higher node similarity. The transition probability for a cross-network random move from a node *u*_*i*_ in ${\mathcal {G}}_{X}$ to a node *v*_*j*_ in ${\mathcal {G}}_{Y}$ is then given by:

5 $$ \Pr\left[{v_{j}|u_{i}}\right]=P_{X \to Y}\left[{i,j}\right]=\frac{1}{{\sum\limits_{\forall v_{j}} {s\left[ {u_{i},v_{j}} \right]} }} \cdot s\left[ {u_{i},v_{j}} \right].   $$

In a matrix form, Eq. (5) can be written as:

6 $$ \textbf{P}_{X \to Y}=\textbf{D}_{S}^{-1}\cdot\textbf{S},  $$

where **S** is a $\left |{\mathcal {U}}\right |\times \left |{\mathcal {V}}\right |$ dimensional matrix for the pairwise node similarity score, and **D**_*S*_ is a $\left |{\mathcal {U}}\right |\times \left |{\mathcal {U}} \right |$ dimensional diagonal matrix such that $D_{S} \left [{i,i}\right ] = \sum \limits _{\forall j}{s \left [ {i,j} \right ]}$. Similarly, the transition probability of a cross-network random move from a node *v*_*i*_ in ${\mathcal {G}}_{Y}\phantom {\dot {i}\!}$ to a node *u*_*j*_ in ${\mathcal {G}}_{X}$ is given by:

7 $$ \Pr \left[u_{j} |v_{i}\right] =P_{Y \to X} \left[{i,j}\right] = \frac{1}{{\sum\limits_{\forall u_{j}} {s^{T}\left[ {v_{i},u_{j}}\right]}}} \cdot s^{T}\left[ {v_{i},u_{j}} \right],   $$

where *s* ^*T*^[*v*_*i*_,*u*_*j*_] is a [*v*_*i*_,*u*_*j*_]-th element of the transposed matrix of **S**. Equation (7) can be written in a matrix form as follows:

8 $$ \textbf{P}_{Y \to X} = \textbf{S}^{T} \cdot \textbf{D}_{S^{T}}^{- 1},  $$

where **S** ^*T*^ is a $\left |{\mathcal {V}}\right |\times \left |{\mathcal {U}}\right |$ dimensional matrix for the pairwise node similarity score, and $ {\textbf {D}_{S^{T}}}\phantom {\dot {i}\!}$ is a $\left | {\mathcal {U}} \right | \times \left |{\mathcal {U}}\right |$ dimensional diagonal matrix such that $D_{S^{T}}\left [{i,i}\right ] = \sum \limits _{\forall j} {s^{T}\left [{i,j}\right ]}$. In fact, the transition probability matrices **P**_*X*→*Y*_ and **P**_*Y*→*X*_ are normalized pairwise node similarity score matrices in the row-wise and column-wise manner.

Finally, we can get the $\left (\left |{\mathcal {U}}\right |+\left |{\mathcal {V}} \right |\right) \times \left (\left |{\mathcal {U}}\right |+\left |{\mathcal {V}}\right |\right)$ dimensional overall transition probability matrix for the Markov random walker over the integrated network *G*, given by

9 $$ \textbf{P} = \left[ \begin{array}{ll} {\textbf{P}_{X}} & {\textbf{P}_{X \to Y}} \\ {\textbf{P}_{Y \to X}} & {\textbf{P}_{Y}} \\ \end{array} \right].   $$

Based on the proposed random walk protocol, the random walker transits more frequently between the pair of nodes (*u*_*i*_,*v*_*j*_) if the node *u*_*i*_ and the node *v*_*j*_ have a higher pairwise node similarity and also if their neighboring nodes also have higher pairwise node similarity (i.e., higher topological similarity). So, as a result, the random walker will spend more time on an edge that connects a pair of nodes (*u*_*i*_,*v*_*j*_), $u_{i} \in {\mathcal {U}}$ and $v_{j} \in {\mathcal {V}}$ as their overall similarity (or node correspondence) increases. Hence, we can effectively estimate the pairwise node alignment probability – which should be proportional to the desired node correspondence – by measuring the steady-state network flow through each edge (*u*_*i*_,*v*_*j*_), $u_{i} \in {\mathcal {U}}$ and $v_{j} \in {\mathcal {V}}$.

**Fig. 4:**
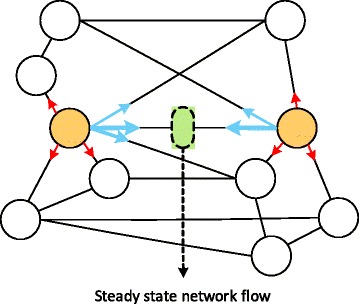
Illustration of the steady-state network flow. Note that the *red colored* arrows indicate the intra-network random moves, while the *blue colored* arrows represent the cross-network random moves

To compute the steady-state network flow, we first compute the steady-state probability *π*(*x*) of the random walker for every node $x \in {\mathcal {U}}\cup {\mathcal {V}} $ in the integrated network. This is equivalent to the long-run proportion of time that the random walker spends at a given node *x*. The steady-state probability distribution is equivalent to the eigenvector of the transition probability matrix **P** that corresponds to unit eigenvalue. This eigenvector, hence the steady-state probability, can be easily obtained through the power method, as the transition probability matrix **P** will be generally sparse for real PPI networks [10, 11]. The steady-state probability *π*(*x*) can be viewed as the amount of ‘water’ at the node *x* in the long-run, and since the amount of the water flow is proportional to the edge weight, we can obtain the steady-state network flow along the edge (*u*_*i*_,*v*_*j*_) as follows (see Fig. 4):

10 $$ \begin{aligned} c\left({u_{i},v_{j}} \right) &= \pi \left({u_{i}} \right) \cdot \Pr \left[ {v_{j} |u_{i}} \right] + \pi \left({v_{j}} \right) \cdot \Pr \left[ {u_{i} |v_{j}} \right] \\ &= \pi \left({u_{i}} \right) \cdot \frac{{s\left({u_{i},v_{j}} \right)}}{{\sum\limits_{\forall v_{j}} {s\left({u_{i},v_{j}} \right)} }} + \pi \left({v_{j}} \right) \cdot \frac{{s\left({u_{i},v_{j}} \right)}}{{\sum\limits_{\forall u_{i}} {s\left({u_{i},v_{j}} \right)} }}. \\ \end{aligned}  $$

This equation can be rewritten in a matrix form as follows:

11 $$ \textbf{C} = \pi_{X} \cdot \textbf{P}_{X \to Y} + \textbf{P}_{Y \to X}^{T} \cdot \pi_{Y},   $$

where **C** is a $\left |{\mathcal {U}}\right |\times \left |{\mathcal {V}}\right |$ dimensional matrix for the steady-state network flow (i.e., pairwise node correspondence scores), *π*_*X*_ is a $\left |{\mathcal {U}}\right |\times \left |{\mathcal {U}}\right |$ dimensional diagonal matrix such that *π*_*X*_[*i*,*i*]=*π*(*u*_*i*_), $u_{i} \in {\mathcal {U}}$, and *π*_*Y*_ is a $\left | \mathcal {V}\right |\times \left |{\mathcal {V}} \right |$ dimensional diagonal matrix such that *π*_*Y*_[*j*,*j*]=*π*(*v*_*j*_), $v_{j} \in {\mathcal {V}}$.

As in SMETANA [10] and SMETANA-CSRW [11], we utilize the following probabilistic consistency transformation (PCT) given by:

12 $$ \tilde{\mathbf{C}} = \alpha \cdot \textbf{C} + \left(1 - \alpha \right) \cdot \textbf{P}_{X} \cdot \textbf{C} \cdot \textbf{P}_{Y}^{T},   $$

to update the estimated node correspondence scores. The above PCT assumes that, given a pair of nodes, if their neighboring nodes have high correspondence, the node pair has increased chance to be aligned. That is, updating the estimated node correspondence score by utilizing the neighbor’s node correspondence could increase the overall accuracy of the node correspondence score. However, the PCT also has the potential risk of creating or increasing false positive node correspondence. That is, some node pairs with zero (or insignificant) correspondence scores can have positive (or increased) node correspondence scores after performing the PCT if they have neighboring nodes with positive correspondence scores, because PCT propagates the node correspondence scores to neighboring nodes. Therefore, to suppress false positive node alignments, we only keep the transformed scores that are larger than the 90 percentile (=*β*). Furthermore, we also keep the original scores *c*[*i*,*j*] even if they are smaller than the threshold *β*. That is,

13 $$ {}\bar{c}\left[i,j\right] = g\left(\tilde{c}\left[{i,j}\right]\right) = \left\{ \begin{array}{ll} \tilde{c}\left[{i,j} \right], & \text{if}~\tilde{c}\left[i,j\right]\geq\beta\text{or } c \left[i,j\right] > 0 \\ 0, & otherwise \\ \end{array} \right..  $$

After transforming and removing node correspondence scores lower than a specific threshold using Eq. (13), we obtain the final node correspondence scores $\bar {\mathbf {C}}$, which will be used to construct the network alignment.



### Constructing the pairwise network alignment

After computing the transformed node correspondence score $\bar {\mathbf {C}}$, we use the scores to construct the network alignment based on the MEA criterion, based on the assumption that the pairwise node alignment probability is proportional to the obtained node correspondence score:

14 $$ \Pr\left[u_{i}\sim v_{j}|\mathcal{G}_{X}, \mathcal{G}_{Y} \right] \propto \bar{c}\left(u_{i},v_{j} \right).  $$

Finally, to find the optimal solution of Eq. (1) based on the derived pairwise node alignment probability, we construct the maximum weighted bipartite matching (MWBM) between $\mathcal {G}_{X}$ and $\mathcal {G}_{Y}$, using an efficient implementation of the MWBM algorithm included in the GAIMC library [23].

## Results and discussion

### Datasets and experimental set-up

We assessed the performance of CUFID-align based on the IsoBase dataset [24], which includes PPI networks of five different species: *H. sapiens* (human), *M. musculus* (mouse), *D. melanogaster* (fly), *C. elegans* (worm), and *S. cerevisiae* (yeast). PPI networks in the IsoBase dataset were constructed by integrating five different databases: BioGRID [25], DIP [26], HPRD [27], MINT [28], and IntAct [29]. In IsoBase, the *H. sapiens* network has 22,369 proteins and 43,757 interactions; the *M. musculus* network has 24,855 proteins and 452 interactions; the *D. melanogaster* network has 14,098 proteins and 26,726 interactions; the *C. elegans* network has 19,756 proteins and 5,853 interactions; and the *S. cerevisiae* network has 6,659 proteins and 38,109 interactions.

We assessed the quality of the predicted network alignment based on the following metrics: correct nodes (CN), specificity (SPE), gene ontology consistency (GOC) scores, conserved interactions (CI), conserved orthologous interactions (COI), and computation time. Note that CN, SPE, and GOC scores assess the biological significance of the alignment, and CI and COI assess the topological quality of the alignment. If the aligned nodes have the same functional annotation based on the KEGG Orthology (KO) group annotations [30], we considered the node alignment to be correct. CN counts the total number of correctly aligned nodes in a given network alignment. SPE is the relative ratio of the total number of correctly aligned node pairs to the total number of aligned node pairs.

To further assess the functional consistency of a given network alignment *A*, we used GOC scores, which can be computed by

15 $$ \begin{aligned} GOC\left(A\right) &= \sum\limits_{\forall \left(u_{i}\sim v_{j}\right)\in A}{goc\left(u_{i},v_{j}\right)} \\&= \sum\limits_{\forall \left(u_{i} \sim v_{j}\right) \in A}\frac{{\left| {GO\left({u_{i}} \right) \cap GO\left({v_{j}} \right)} \right|}}{{\left| {GO\left({u_{i}} \right) \cup GO\left({v_{j}} \right)} \right|}}, \end{aligned}  $$

where *G* *O*(*x*) denotes the set of all GO terms assigned to the protein *x*. To compute the GOC scores, we downloaded the latest version of GO annotations for each species from GO consortium [31] (Feb. 10, 2016 version). We only used GO terms that have experimental evidence (i.e., those that include the codes ‘EXP’, ‘IDA’, ‘IPI’, ‘IMP’, ‘IGI’, and ‘IEP’). Additionally, similar to [32], we removed every GO term whose information content (IC) was smaller than 2, in order to compute GOC scores based on more informative GO annotations. IC is defined as

16 $$ IC\left(c\right) = - \log_{2}\frac{{\left| c \right|}}{{\left| {root\left(c \right)} \right|}},  $$

where |*c*| is the number of proteins having the particular GO term *c*, and |*r* *o* *o* *t*(*c*)| is the total number of proteins under the root GO term of the particular GO term *c*, where three root GO terms are molecular function (MF, GO:0003674), biological process (BP, GO:0008150), and cellular component (CC, GO:0005575). Note that if at least one protein in the aligned protein pair does not have a functional annotation such as KO group annotations or GO terms, the aligned protein pair was removed before computing the performance metrics CN, SPE, and GOC scores.

To assess the topological quality of the constructed network alignment, we counted the number of conserved interactions (CI) as follows:

17 $$ \sum\limits_{\forall\left({u_{i}, u_{j}}\right) \in \mathcal{D}}\mathbf{1}\left[\left({u_{i},u_{j}} \right) \in {\mathcal{D}} \right] \cdot {\mathbf{1}}\left[(f\left({u_{i}} \right),f\left({v_{j}} \right)) \in \mathcal{E}\right],  $$

where **1**[·] is the indicator function whose value is 1 if the statement in the bracket is true and 0 otherwise, and *f*(*x*) denotes the corresponding protein aligned to the protein *x*. However, the conserved interactions may not be necessarily be significant from a biological perspective if the aligned proteins connected by the conserved interactions are not orthologous. Considering the large size of typical PPI networks, simply aiming at a network alignment that maximizes the number of conserved interactions may risk overfitting the network topology without clear biological significance, which can be especially problematic when PPI networks are incomplete and noisy. For this reason, in order to assess the biological significance of the topological mapping in a given network alignment, we counted the number of conserved orthologous interactions, which is the number of conserved interactions between orthologous protein pairs (COI). This is given by:

18 $$ \sum\limits_{\forall\left(u_{i},u_{j}\right) \in {\mathcal{D}}}h\left({u_{i},u_{j}} \right) \cdot {\mathbf{1}}\left[{\left({u_{i},u_{j}} \right) \in {\mathcal{D}}}\right] \cdot {\mathbf{1}}\left[(f\left({u_{i}} \right),f\left({u_{j}} \right)) \in {\mathcal{E}}\right],  $$

where

19 $$ h\left({u_{i},u_{j}} \right) = \left\{\begin{array}{ll} 1, & \text{if}~\left[goc\left(u_{i}, f\left({u_{i}}\right)\right) \cdot goc\left({u_{j},f\left({u_{j}} \right)} \right) \right] > 0\\ {0,} & otherwise\\ \end{array} \right..  $$

We compared the performance of CUFID-align against a number of state-of-the-art alignment methods: IsoRank [6], SMETANA [10], SMETANA-CSRW [11], PINALOG [13], and HubAlign [14]. Additionally, to verify the effectiveness of the network-based approach over the conventional approach that uses sequence similarity alone, we compared the various network-based methods and with a method that finds the best mapping between networks solely based on the sequence similarity between the proteins. More specifically, given a network pair, we tried to predict the network alignment by using maximum weighted bipartite matching based on BLAST bit scores. Since both SMETANA and SMETANA-CSRW yield many-to-many mappings by default, we used the parameter *n*_*max*_=1 to obtain one-to-one mappings. Other than this, the default parameters were used in our experiments (i.e., *α*=0.9 and *β*=0.8). For HubAlign, we used the default parameters (i.e., *λ*=0.1, *d*=10, and *α*=0.7). For IsoRank, we set the parameter *α*=0.6 as recommend in the original paper. For CUFID-align, we set the parameter *α*=0.9 and *β*=90 percentile of the transformed correspondence score. We performed all experiments on a desktop computer equipped with a 3.2 GHz Intel i5 quad-core processor and 8 GB memory.

### Performance assessment based on the IsoBase dataset

We assessed the performance of CUFID-align by predicting the alignment for every pair of PPI networks in the IsoBase dataset. CN and SPE are summarized in Table 1. As we can see, CUFID-align and BLAST-MWBM achieve higher CN in all test cases. This means that CUFID-align and BLAST-MWBM can generally align a larger number of proteins that have the same functional annotations (*i.e.*, KEGG orthologous group annotations) than the other state-of-the-art network alignment methods. Interestingly, the sequence-similarity-based approach can identify a larger number of correct nodes (CN) than most of the other network-based approaches. However, as will be shown later, it is clearly biased and the method performs very poorly in terms of the topological quality of the predicted network alignment. CN for PINALOG and HubAlign may depend on the average degrees of the PPI networks (i.e., $\left |{\mathcal {E}}\right |\left /\left | {\mathcal {V}} \right |\right.$). That is, if one of the PPI networks has a much lower average degree, the overall quality of the network alignment may be significantly degraded. Note that human, yeast, and fly PPI networks have relatively higher average degrees, and mouse and worm PPI networks have relatively lower average degrees. Since PINALOG and HubAlign adopt a seed-and-extension approach, the search space for aligning addition protein pairs is restricted to the neighboring nodes of the seed network. Hence, it would be possible that PINALOG and HubAlign may align proteins even though there is no orthologous protein pair in the search space (i.e., the current set of neighboring nodes), which may affect the quality of the final alignment.

**Table 1:** Pairwise alignment results for the IsoBase dataset. Protein functionality is determined based on the KEGG Orthology (KO) group annotations

	Yeast – Fly		Yeast – Worm		Yeast – Human		Yeast – Mouse		Fly – Worm	
	CN ^a^	SPE ^b^	CN	SPE	CN	SPE	CN	SPE	CN	SPE
CUFID-align	1,708	0.748	**1,548**	0.834	1,330	0.736	**1,304**	0.794	2,616	0.873
SMETANA-CSRW	1,610	0.757	1,426	**0.850**	1,224	0.733	1,192	**0.802**	2,444	0.870
SMETANA	1,530	0.733	1,422	0.843	1,134	0.710	1,182	0.782	2,338	0.852
PINALOG	1,368	0.722	640	0.737	1,100	0.682	76	0.400	672	0.689
HubAlign	1,326	0.681	98	0.170	1,082	0.633	42	0.231	102	0.201
IsoRank	1,414	0.712	650	0.703	1,142	0.702	76	0.369	918	0.818
BLAST-MWBM^c^	**1,712**	**0.776**	1,544	0.836	**1,334**	**0.768**	1,280	0.792	**2,680**	**0.885**
	Fly – Human		Fly – Mouse		Worm – Mouse		Worm – Human		Human – Mouse	
	CN	SPE	CN	SPE	CN	SPE	CN	SPE	CN	SPE
CUFID-align	2,528	0.754	2,364	**0.788**	1,818	0.807	1,858	0.791	**5,178**	**0.983**
SMETANA-CSRW	2,358	0.763	2,146	0.768	1,610	**0.811**	1,722	**0.803**	5,002	0.978
SMETANA	2,096	0.706	2,112	0.764	1,578	0.803	1,570	0.780	4,876	0.972
PINALOG	1,172	0.604	118	0.567	66	0.458	482	0.677	282	0.972
HubAlign	354	0.219	34	0.230	24	0.188	32	0.063	144	0.667
IsoRank	1,736	0.725	146	0.566	72	0.456	644	0.793	286	0.979
BLAST-MWBM	**2,580**	**0.766**	**2,374**	0.781	**1,824**	0.808	**1,884**	0.794	5,140	0.982

^a^CN: ccorrect nodes

^b^SPE: specificity

^c^BLAST-MWBM: maximum weighted bipartite matching of PPI networks only using the BLAST bit score

In each column, the best performance is shown in boldface

When it comes to the specificity of the alignment results, random walk based methods (CUFID-align, SMETANA-CSRW, and SMETANA) achieve relatively higher SPE compared to PINALOG and HubAlign. SPE of HubAlign appears to be more sensitive than the other methods with respect to the average degrees of the PPI networks. CUFID-align, SMETANA-CSRW, and SMETANA achieve similar SPE, often higher than those of PINALOG and HubAlign. This means that CUFID-align can in general more accurately align protein pairs that have the same functional annotations compared to PINALOG and HubAlign.

**Fig. 5:**
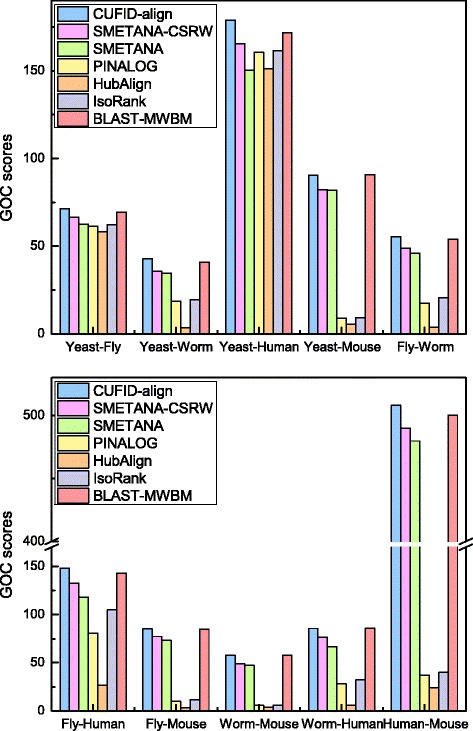
GOC scores of various pairwise network alignment algorithms

Since proteins can have multiple functions, we further evaluated the functional consistency of the alignment results based on the GOC scores, where higher GOC scores indicate that the obtained alignments are functionally more coherent. As we can see in Fig. 5, CUFID-align achieves higher GOC scores than the other compared algorithms in all test cases. Again, if the network pairs have higher average degrees, PINALOG and HubAlign show comparable GOC scores. However, probably due to the restricted search space of the seed-and-extend approach, GOC scores of PINALOG and HubAlign tend to be smaller than the other methods when the average degree of one of the PPI networks is relatively smaller than that of the other. In comparison, CUFID-align is more robust to the change of topological properties such as the average degrees of the PPI networks to be aligned.

The above results show that CUFID-align can accurately predict matching proteins in different species that have similar functionalities, according to the functional annotations of proteins that are currently available. The results also imply that the proposed algorithm may provide a useful tool for predicting the functions of unknown proteins in less studied species through network alignment with species that have been better studied.

Next, to assess the topological quality of the network alignment results, we compared the number of conserved interactions (CI) predicted by different methods. Table 2 shows the CI for all compared methods. As we can see in Table 2, CUFID-align can identify a larger number of conserved interactions than SMETANA-CSRW and SMETANA, but it is smaller than HubAlign and PINALOG. In fact, our results show that PINALOG and HubAlign outperform the other methods in terms of CI. One interesting observation is that although PINALOG and HubAlign can identify a large number of conserved interactions compared to CUFID-align, GOC scores for PINALOG and HubAlign are much smaller than CUFID-align as shown in Fig. 5. Since both PINALOG and HubAlign adopt a seed-and-extension approach, the algorithms only align protein nodes if they are connected to the seed network alignment. PINALOG and HubAlign may have a higher risk for overfitting the prediction outcomes to the topological structure of the PPI networks compared to the other methods, and they may not as effectively deal with the inserted or deleted nodes as the random walk based methods, which may be problematic when handling PPI networks that are incomplete and/or contain many errors (e.g., many false positive interactions). As the GOC scores were low for PINALOG and HubAlign, despite the high CI they attained, we wanted to further evaluate the biological significance of the conserved interactions in the predicted network alignment results. For this purpose, we counted the number of conserved interactions between orthologous protein pairs. Table 3 summarizes the number of conserved orthologous interactions (COI) predicted by different algorithms. Note that, for this experiment, we did not consider the alignment of networks whose average degrees differ significantly, since there will be only a small number of conserved orthologous interactions in such cases. Table 3 shows that CUFID-align achieves comparable or higher COI compared to PINALOG and HubAlign except for the alignment between the yeast and human PPI networks.

**Table 2:** Number of conserved interactions (CI) obtained by different network alignment algorithms

	Yeast – Fly	Yeast – Worm	Yeast – Human	Yeast – Mouse	Fly – Worm
CUFID-align	1,721	486	3,421	56	347
SMETANA-CSRW	337	110	2,468	31	107
SMETANA	504	171	2,377	37	116
PINALOG	2,982	1,000	6,231	225	666
HubAlign	836	4,013	2,659	545	3,276
IsoRank	1,436	764	3,165	176	558
BLAST-MWBM	246	89	1,317	14	70
	Fly – Human	Fly – Mouse	Worm – Mouse	Worm – Human	Human – Mouse
CUFID-align	1,547	59	18	459	318
SMETANA-CSRW	710	41	8	198	336
SMETANA	965	50	16	283	337
PINALOG	2,730	88	47	917	358
HubAlign	9,317	491	459	3,743	532
IsoRank	1,471	106	130	569	350
BLAST-MWBM	441	12	2	138	253

**Table 3:** Number of conserved orthologous interactions (COI) obtained by different network alignment algorithms

	Yeast – Fly	Yeast – Worm	Yeast – Human	Yeast – Mouse	Fly – Worm
CUFID-align	91	10	743	5	3
SMETANA-CSRW	91	8	749	6	2
SMETANA	86	10	705	8	4
PINALOG	129	15	970	19	4
HubAlign	57	2	634	15	5
IsoRank	94	11	741	10	4
BLAST-MWBM	74	8	556	4	2
	Fly – Human	Fly – Mouse	Worm – Mouse	Worm – Human	Human – Mouse
CUFID-align	202	13	1	21	111
SMETANA-CSRW	196	10	1	26	139
SMETANA	230	14	1	26	123
PINALOG	180	22	2	27	134
HubAlign	67	15	4	5	98
IsoRank	185	17	1	18	142
BLAST-MWBM	112	6	0	15	94

We also compared the network-based approaches with the sequence-similarity-based approach. As we can see in Table 1 and Fig. 5, a simple sequence-similarity-based approach can construct network alignments with high functional coherence, and that the node similarity score may provide useful guidelines for identifying orthologous proteins. However, this results should be taken with a grain of salt, since they are likely due to the fact that the current functional annotations of proteins are often based on sequence similarity between proteins. As shown in Tables 2 and 3, BLAST-MWBM – which uses BLAST bit score and MWBM without using any network information – can identify a much smaller number of CIs and COIs compared to the network-based methods. These results imply that strong dependence on sequence similarity for constructing a network alignment has the potential risk of getting biased results that may fail to capture important protein interactions that are conserved across different species, which may be critical in deciphering the underlying cellular mechanisms that involve those interactions. In contrast, network-based methods, including CUFID-align, that incorporate topological information for constructing network alignments can make accurate and balanced predictions that identify both orthologous proteins as well as conserved interactions. Our results clearly show the importance of effective integration of node similarity and topological similarity for effective comparative analysis of PPI networks.

Finally, Table 4 shows the computation time for each method. As we can see in this table, CUFID-align needs the least computation time among all compared methods in most test cases. Computation time of HubAlign largely depends on the average degrees of the PPI networks because HubAlign takes a seed-and-extension approach, whose search space is strongly affected by the average degrees of the PPI networks to be aligned. Computation time of SMETANA-CSRW is proportional to the size of the PPI networks. The bottleneck for SMETANA-CSRW is the step for constructing the transition probability matrix of the context-sensitive random walker (CSRW), whose computation time is proportional to the size of the two PPI networks that need to be aligned. PINALOG requires a relatively long computation time compared to other methods in most cases, as shown in Table 4.

**Table 4:** CPU time of the tested network alignment algorithms (in seconds)

	Yeast – Fly	Yeast – Worm	Yeast – Human	Yeast – Mouse	Fly – Worm
CUFID-align	6.22	4.79	11.22	5.70	12.88
SMETANA-CSRW	243.64	163.24	448.29	435.94	3,002.20
SMETANA	6.65	5.81	11.47	9.12	26.11
HubAlign	451.24	75.67	571.23	5.30	55.87
PINALOG	997.85	1,654.66	1,984.03	2,202.15	2,141.00
IsoRank	1,737.07	369.52	3401.29	64.47	181.55
	Fly – Human	Fly – Mouse	Worm – Mouse	Worm – Human	Human – Mouse
CUFID-align	18.93	18.38	25.71	28.36	68.59
SMETANA-CSRW	6,104.70	6,420.80	6,383.70	6,084.10	4,9185.00
SMETANA	63.43	60.85	53.24	56.28	454.11
HubAlign	532.31	4.92	1.91	84.45	8.99
PINALOG	3,127.35	1,611.39	101.86	6,764.56	4,864.16
IsoRank	1433.27	37.77	16.92	326.79	77.64

### Extension of CUFID-align for the alignment of multiple networks

In this work, we have focused on the steady-state network flow approach and its application to the pairwise network alignment problem. However, the problem of multiple network alignment has been gaining wide interest in the research community and its practical importance has been increasing as the number of available PPI networks for different species continue to increase. Although it is beyond the scope of the current paper, we expect the extension of CUFID-align for multiple network alignment will be relatively straightforward. First of all, to this aim, we can modify the transition probability matrix in Eq. (9) by concatenating the normalized adjacency matrices and node similarity score matrices for the multiple PPI networks to be aligned. Following the construction of this extended transition probability matrix, the steps for computing the node correspondence scores – shown in Eqs. (11) and (13) – can be modified by constructing diagonal matrices and inserting corresponding the matrices into the diagonal terms. The extended version of CUFID-align for multiple PPI network alignment is expected to have distinctive advantages over other existing multiple PPI network alignment algorithms. First, it may be able to estimate the ‘global’ node correspondence scores more accurately. Currently, most multiple PPI network alignment algorithms estimate the node correspondence scores for every PPI network pair in the interest of computational complexity. The estimated pairwise node correspondence scores are later updated based on additional transformations to make them more suitable for multiple network alignment. However, considering that the ultimate goal is in constructing the alignment of multiple networks, it would be preferable to estimate the node correspondence scores (or equivalently, node alignment probabilities) Pr[*u*_*i*_∼*v*_*j*_|**G**] considering all networks, rather than just estimating $\Pr \left [ {u_{i} \sim v_{j} |{\mathcal {G}}_{X},{\mathcal {G}}_{Y}} \right ]$ based on the given network pair, where $u_{i} \in {\mathcal {G}}_{X} $, $v_{j} \in {\mathcal {G}}_{Y} $, and **G** is the set of all PPI networks including ${\mathcal {G}}_{X}$ and ${\mathcal {G}}_{Y}$. Since the aforementioned extension of CUFID-align estimates the node correspondence scores based on an integrated network that combines all networks in **G**, it has the potential to accurately compute the posterior node-to-node alignment probability given all the networks. Computation of such ‘global’ node correspondence score may lead to improved multiple network alignment results. Second, the extended version of CUFID-align will still be computationally very efficient, as most steps in CUFID-align only require simple matrix operations even if extended to multiple networks. Finally, the extended approach will require relatively low computational resources (especially, in terms of memory). For example, suppose that there are *N* PPI networks, where the number of nodes in the *i*-th network *G*_*i*_ is *V*_*i*_. To align the *N* PPI networks, IsoRankN will need the pairwise node correspondence scores for each of the $\binom {N}{2}$ network pairs, where for each pair, the algorithm will need to construct a |*V*_*i*_·*V*_*j*_|×|*V*_*i*_·*V*_*j*_| dimensional matrix. However, CUFID-align can compute the global node correspondence scores by constructing a single $\left | {\sum \limits _{i = 1}^{N} {V_{i}} } \right | \times \left | {\sum \limits _{i = 1}^{N} {V_{i}}}\right |$ dimensional matrix. We are currently working on extending CUFID-align for multiple network alignment.

## Conclusions

In this paper, we proposed CUFID-align, a novel network alignment algorithm based on the concept of steady-state network flow of a Markov random walk model on an integrated network. Given a pair of PPI networks, CUFID-align constructs an integrated network and a Markov random walk model on the resulting network such that the steady-state network flow between a pair of nodes in different PPI networks increases when the nodes have higher pairwise node similarity (typically measured based on sequence similarity) and topological similarity. For this purpose, the Markov random walk model is designed to make more frequent transitions between protein nodes that have higher overall similarity, thereby making the steady-state network flow – which reflects the long-run behavior of the random walker – an effective measure of the correspondence between nodes that belong to different networks. As we have shown in our performance assessment results using real PPI networks in the IsoBase database, CUFID-align can accurately align proteins with identical functional annotations at a relatively low computational cost. Our results show that CUFID-align may provide an effective means of computationally annotating the functions of proteins through comparative analysis of PPI networks.
